# The values of neutrophil to lymphocyte ratio and platelet to lymphocyte ratio in predicting 30 day mortality in patients with acute pulmonary embolism

**DOI:** 10.1186/s12872-016-0304-5

**Published:** 2016-06-04

**Authors:** Yaqing Ma, Yimin Mao, Xuegai He, Yuxia Sun, Shenshen Huang, Jiayong Qiu

**Affiliations:** Department of Respiratory Medicine, The First Affiliated Hospital, and College of Clinical Medicine of Henan University of Science and Technology, 471003 Luoyang, China

**Keywords:** Pulmonary embolism, Neutrophil, Lymphocyte

## Abstract

**Background:**

vAcute pulmonary embolism (PE) is a life threatening disease. The treatment options depend on the severity of the disease and the mortality varies widely depending on the severity of the condition. It is important to identify patients who are at high risk of mortality. The aim of the present study was to explore the prognostic alues of neutrophil to lymphocyte ratio (NLR) and platelet to lymphocyte ratio (PLR) for 30-day mortality in patients with acute PE.

**Methods:**

The study included 321 patients admitted to our university hospital between January 2013 and May 2015 with the diagnosis of acute PE. Multivariable risk models were developed to assess the predictive values of the NLR and PLR for 30-day mortality. Discrimination was evaluated using receiver operating characteristic (ROC) curves.

**Results:**

Two hundred forty-eight patients met our selection criteria. Twenty of them died within 30 days of hospital admission. NLR was found to be an independent predicator after other confounding factors were adjusted in the model. For 1 unit of increase of NLR, the risk of 30-day mortality rose about 13  % (OR = 1.13,95 % CI: 1.04–1.23). The area under ROC for NLR is 0.79 (95 %CI: 0.703–0.880). PLR was associated with 30-day mortality in univariate analysis but the predicative ability diminished with inclusion of other predicators in multivariable model.

**Conclusions:**

NLR is readily available predicator for short-term mortality. It could be a useful indicator for identifying high risk population and guiding clinical management of acute PE.

## Background

Acute pulmonary embolism (PE) is a serious disease associated with high mortality rates despite advanced therapeutic options. It is estimated that the incidence rates of PE is about 23 to 69 cases per 100,000 persons annually in the United States [1]. PE accounts for 300,000 deaths in Europe alone each year [2]. The short-term mortality varies widely from 2 to 95  %, depending on the severity of the disease [3]. It is important to identify prognostic markers for those who are at greater risk of short-term death. In 2014, the European Society of Cardiology classified the severity of acute PE based on the estimated PE-related early mortality risk. Patients with suspected or confirmed shock or persistent arterial hypotension are classified as high risk. Patients without these clinical status are classified as “not high risk” [4]. The classification has important implications for PE diagnosis and treatment options. It is recommended to use anti-coagulation treatment for low risk patients, thrombolytic agents or embolectomy for high risk patients. However, there is no explicit clinical criterion regarding the use of anticoagulation or thrombolysis for patients with intermediate risk of PE. Meyer and colleagues [5] conducted a randomized, double-blind trial to compare treatment effects of anticoagulation and thrombolysis for patients with intermediate-risk of PE. It was found that fibrinolytic therapy prevented hemodynamic instability but increased the risk of hemorrhage and stroke. Sharifi discovered that low-dose tissue plasminogen activator is safe and effective in the treatment of intermediate-risk PE and could reduce the pulmonary artery pressure for more than half year [6]. We believe that the accurate prediction of high risk PE would help clinicians to identify appropriate patients for thrombolytic therapy.

Many prognostic indicators have been developed for PE, such as the simplified pulmonary embolism severity index (sPESI), brain natriuretic peptide (BNP), N-terminal pro-Brain Natriuretic Peptide (NT-pro-BNP), indications of right ventricular dysfunction, heart-type fatty acid-binding protein, and troponin concentrations. However, findings for most of these indicators are inconsistent and many of them are difficult to apply in the clinical setting or not measured on routine basis, especially for developing countries where resources are limited.

Virchow [7] was first to describe that thrombus formation resulted from abnormalities in three areas including blood flow, the vessel wall, and blood components. It is well known that inflammation and thrombosis are interrelated. Inflammation can cause endothelial damage, increase procoagulant factors, inhibit natural anticoagulant pathways and fibrinolytic activity [8]. Moreover, severe hypoxia as result of blockage of pulmonary arteries could increase activity of neurohormonal and adrenergic system and induce the release of inflammatory cytokine. Such response could exacerbate thrombosis and patients’ condition [9]. Kurtipek found the association between NLR and PLR values and endothelial dysfunction in patients with PE. It is postulated that the endothelial dysfunction could play a role in the development of cardiovascular events for PE patients [10].

Neutrophil to lymphocyte ratio (NLR) and platelet to lymphocyte ratio (PLR) are found to be associated with many inflammatory diseases. These indicators have been used increasingly as new prognostic markers for risk predication of cardiovascular disease and cancers [11–14]. Limited studies have explored the predicative abilities of NLR and PLR on short-term mortality among patients with acute PE [11, 15, 16]. However, some important confounding was not controlled in analysis and the predicative abilities of NLR and PLR was not fully assessed. Furthermore, NLR and PLR values are influenced by many factors and the predictive ability could be confounded by these factors. It is also important to exclude patients with NLR or PLR-related diseases to avoid the interference of disease status on NLR and PLR, such as severe infection and hematological diseases. The prognostic values of these indicators remain to be further evaluated. Accurate risk stratification of acute PE are critical important to determine prognosis. The identification of novel prognostic factors would help to guide interventions and avoid side effects from inappropriate treatment. Both NLR and PLR are readily available indicators during routine laboratory tests. The prognostic values of these indicators would allow active monitoring of disease progress and guide disease management among PE patients. The objective of the current study is to explore the values of NLR and PLR in predicating 30-days mortality in patients with acute PE.

## Methods

We identified 357 adult patients with diagnosis of acute PE at the First Affiliated Hospital of HeNan University of Science and Technology, Henan, China, between January 2013 and May 2015. Thirty-six patients were lost-to-follow up. A total of 321 patients were included in this study. The diagnosis of PE was based on CT pulmonary angiogram (CTPA) and suspected cases were excluded from the study.

The 30 day-mortality was identified through medical records or contacting patients or their relatives regarding the status of survival or date of death. Patients who died within 30 days of hospital admission were defined as short-term death.

Patients were excluded if they (1) received anticoagulation or thrombolysis treatment before their blood test; (2) or if they met one of the following conditions: a) hematological diseases; b) diagnosed with any other disease that could affect blood cell count (WBC > 20 × 10^9^/L or WBC < 3 × 10^9^/L; Hgb < 80 g/L; Platelet < 80 × 10^9^/L), such as patients with clear evidence of active infection; c) had blood transfusion during the past 2 weeks; d) received immunosuppressant or steroid drugs in the last 2 weeks; e) had acute coronary syndrome; e) advanced liver or renal disease. Two hundred forty-eight patients met our inclusion criteria.

Data was extracted from the hospital electronic database, including patient characteristics, medical tests within 24 h of hospital admission, and CTPA and echocardiograph performed during the last 72 h of hospital admission. sPESI was calculated based on age (≥80), history of cancer, history of chronic cardiopulmonary disease/heart failure, systolic blood pressure <100 mmHg, heart rate ≥110 bpm, and arterial oxygen saturation <90  %. RVD was diagnosed by ultrasonic cardiography (UCG-RVD) with the following criteria (1) right ventricular and left ventricular diameter ratio >1; right ventricular free wall motion amplitude < 5 mm, and (3) leftward shift of the ventricular septum [17]. The CT images of right ventricular dysfunction included the axial diameter of the RV being larger than the diameter of the left ventricular.

The statistical analysis was done using SPSS v.21. The normality of distribution for continuous data was determined by Kolmogorov-Smirnov test. Student t test was used to compare two groups of normally distributed data, and Mann–Whitney U test was employed for no normally distributed data. *x*^2^ test or Fisher’s Exact test were performed for categorical data.

Forward logistic regression was applied to determine the association between NLR or PLR and short-term mortality, while controlling for potential confounding. Hosmer-Lemeshow goodness-of-fit test was employed to assess the precision of the predictive probabilities generated by the predicative model. It assesses whether or not the observed mortality match expected mortality based on expected probabilities. A *p* value > 0.05 indicated acceptable predicative precision for each model.

The predicative accuracies of NLR and PLR were determined through receiver operating characteristic curves (ROC). The area under the ROC curve reflects how good the test is at discriminating between patients with or without short-term death. A value >0.7 confirmed the usefulness of an indicator in predicting 30-daydeath. ROC was used to determine the best cut-off points for prediction of 30-day mortality.

## Results

Seventy-three patients were excluded because they didn’t meet our criteria for blood cell count (32 cases), had unqualified echocardiograph (31 cases), received blood transfusion (4 cases), had hematological diseases (3 cases), or received immunosuppressant or steroid drugs (3 cases). A total of 248 patients met the selection criteria and were included in the study. Twenty patients died within 30 days of hospital admission. The characteristics of the study population are presented in Table 1. Patients who died within 30-days of admission are more likely to be older, have history of operation or bed rest immobilization within the last 4 weeks, have higher heart or respiratory rate, right ventricular dysfunction, higher systolic blood pressure, sPESI, BNP, neutrophil, NLR, red cell distribution width (RDW), and PLR. The lymphocyte (LYM), hemoglobin (HGB), and hematocrit (HCT) were significantly lower among patients with short-term morality (Table 1).

**Table 1:** Demographical and clinical characteristics of the study patients

	Survivors (*n* = 228)	Deaths (*n* = 20)	*P*
Age (year)	66 (52–74)	75 (65–83)	0.001
Sex (Male)	126 (55.3 %)	13 (65 %)	0.400
Coronary heart disease	59 (25.9 %)	4 (20 %)	0.563
Diabetes	23 (10.1 %)	3 (15 %)	0.759
Hypertension	74 (32.5 %)	7 (35 %)	0.816
COPD	43 (18.9 %)	7 (35 %)	0.151
Chronic heart failure	41 (18.0 %)	5 (25 %)	0.984
Cancer	14 (6.1 %)	4 (20 %)	0.066
Had operation or bed rest immobilization <4 weeks	54 (23.7 %)	10 (50 %)	0.010
T (°C)	36.7 (36.4–37.1)	36.6 (36.4–36.8)	0.237
RR (min)	21 (20–23)	23 (20–25)	0.007
HR (min)	88 (80–96)	94 (86–108)	0.007
SBP (mmHg)	124 (110–140)	131 (121–150)	0.023
BNP (pg/ml)	209.25 (52.35–835.475)	1709.1 (676.1–4986)	0.000
LgBNP	2.29 ± 0.85	3.19 ± 0.66	0.000
UCG-RVD	70 (30.7 %)	12 (60 %)	0.008
CT-RVD	61 (26.8 %)	10 (50 %)	0.027
sPESI	0 (0–1)	1 (1–2)	0.000
WBC (10^9^/L)	8.47 ± 3.05	9.146 ± 3.24	0.348
NEU (10^9^/L)	5.70 (4.36–7.96)	7.79 (5.58–9.15)	0.035
LYM (10^9^/L)	1.41 ± 0.71	0.74 ± 0.33	0.000
Monocytes (10^9^/L)	0.46 (0.29–0.60)	0.37 (0.24–0.55)	0.285
HGB (g/L)	126.145 ± 19.11	111.05 ± 17.61	0.001
HCT	38.1 ± 5.49	33.7 ± 5.29	0.001
MCV	91.55 (88.10–94.95)	91.70 (89.35–93.83)	0.823
RDW (fl)	44.35 (41.6–49.0)	47.0 (45.0–50.5)	0.033
PLT (10^9^/L)	228.5 (182.25–298.0)	228.5 (172.25–300.20)	0.868
MPV (fl)	9.26 ± 1.10	9.02 ± 1.046	0.346
PDW (fl)	16.1 (15.83–16.40)	16.00 (15.90–16.50)	0.967
NLR	4.43 (2.83–7.88)	10.00 (6.13–18.49)	0.000
PLR	172.25 (125.19–284.5)	371.94 (221.13–511.37)	0.000

*COPD* Chronic Obstructive Pulmonary Disease, *T* Body Temperature, *RR* Respiratory Rate, *HR* Heart Rate, *SBP* Systolic Blood Pressure, *BNP* Brain Natriuretic Peptide, *UCG-RVD* Right Ventricular Dysfunction Diagnosed by Ultrasonic Cardiography, *CT-RVD* Right Ventricular Dysfunction Diagnosed by CT, *WBC* White Blood Cells, *NEU* Neutrophil, *LYM* Lymphocyte, *HGB* Hemoglobin, *HCT* Hematocrit, *MCV* Mean Corpuscular Volume, *RDW* Red Cell Distribution Width, *PLT* Platelets, *MPV* Mean Platelet Volume, *PDW* Mean Platelet Volumn, *NLR* Neutrophil to Lymphocyte Ratio, *PLR* Platelet to Lymphocyte Ratio

As the calculation of sPESI includes age, heart rate, and systolic blood pressure, those risk factors were excluded from the risk models to avoid collinearity. BNP was log transferred in the logistic regression as the distribution of BNP was highly skewed. The results of the multivariate analysis show that NLR is an independent predicator for 30-day mortality among patients with acute PE. The probability of death increased about 13.2  % (OR = 1.13, 95 % CI: 1.04–1.23) with one unit of increase of NLR (Table 2). LgBNP, sPESI, and HGB were also significant predicators of 30-day mortality for PE patients. The risk model has a good calibration with *p* value of 0.721.

**Table 2:** Multivariate regression results of short-term death for acute pulmonary embolism

Risk factors	OR	95 % CI		*P*
		Lower	Upper	
NLR	1.132	1.044	1.228	0.003
HGB	0.942	0.907	0.979	0.003
LgBNP	2.913	1.247	6.804	0.014
sPESI Goodness-of-fit test (p)	1.936	1.121	3.345	0.018 0.721

*OR* odds ratio, *CI* confidence interval

PLR is no longer a predictor in multivariate analysis after other predicators were included in the model (OR = 1.002, 95  % CI: 0.997–1.008).

The areas under ROC is 0.792 (95 % CI: 0.703–0.880, *P* < 0.001) for NLR and 0.789 (95 % CI:0.699–0.880, *P* < 0.001) for PLR. The cut-off value of 5.99 for NLR had sensitivity and specificity values of 80  % and 66.7  %. The sensitivity and specificity were 65 and 80.7  % for a cut-off of 325.0 for PLR (Table 3).

**Table 3:** Values of area under the ROC curve (AUC), sensitivity and specificity for the optimal cut-off point of NLR and PLR for predicting short-term death among patients with acute pulmonary embolism

Predicators	AUC (95 % CI)	*P*	Cut-off	Sensitivity	Specificity
NLR	0.792 (0.703–0.880)	0.000	5.99	80 %	66.70 %
PLR	0.789 (0.699–0.880)	0.000	325.0	65 %	80.70 %

## Discussion

NLR represents the balance between neutrophils and lymphocytes. It has been proposed as a new marker for systemic inflammation [18]. Previous studies found that increased NLR is associated with cardiovascular mortality [19]. The high NLR is results of increased neutrophil count and decreased lymphocyte count. The elevated serum cortisol levels, as a response to stress, may account for such change. The inflammation procedure could stimulate the production of neutrophil and speed up the apoptosis of lymphocytes. Moreover, the increased number of neutrophils is associated with inflammatory process which could contribute to thrombotic state. Jo found that systemic inflammatory response syndrome criteria were significantly more common for PE patients who died within 30 days of hospital admission [20]. It reveals the role of systemic inflammation for short-term mortality among PE patients. The results of this study confirm the hypothesis that NLR is a significant risk predictor of 30-day mortality. It was observed that increased neutrophil and decreased lymphocyte count within deceased group. The NLR had good discrimination and calibration for short-term morality, demonstrating its usefulness in predicating short-term mortality. The predicative ability was independent of many other predicators included in the model. The result of our study is consistent with recently finding, which indicates that NLR is useful for risk stratification in patients with venous thromboembolism [21]. The risk of short-term death was increased by about 13  % with increase of every unit of NLR. By evaluating NLR, clinicians may identify high risk patients among those who would be classified as low risk or intermediate risk on the basis of other predicators. Treatment plans can be adjusted based on calculated NLR to reduce the possibility of short-term death. In addition, NLR is measured on routine basis. It has potential to be used to monitor the disease progress for PE patients.

Recently, PLR has been considered as a new marker of systemic inflammation [22]. It was found that PLR has better prognostic value than platelet or lymphocytes alone in predicting certain cardiovascular diseases [23]. We found that PLR was significantly higher for patients who died within 30-days of hospital admission in the univariate analysis. However, the predictive ability diminished after confounding factors were included in the model. One study found that PLR was significantly correlated with sPESI [24]. It indicates that the prognostic value observed in univariate analysis may not be independent predicator for 30-day mortality. In addition, the sensitivity of PLR at optimal cut-off point is relatively low considering the severity of the complication. PLR might not be a good predicator for short-term mortality.

RDW was found to be a diagnostic measure for patient with suspected PE and it could aid in the risk stratification of patients with PE [9]. The results of our study show that predictability of NLR is not confounded by RDW. It is consistent with their finding that RDW is not corrected with NLR. NLR could add additional value in predicating short-term mortality for patients with PE.

## Limitations

This study had some limitations. This is a single center retrospective study with a limited sample size. For some patients, it is difficult to differentiate PE with pulmonary infection. The results of this study results could be biased by such misclassification. Troponin is considered an important predictor for PE. This indicator was not included in our analysis, as this indicator was not performed for about half of the study participants. Some PE patients might have unstable COPD. The severity of COPD is associated with PE as well as death. Our data doesn’t contain detailed classification of COPD. Although COPD was controlled in multivariate analysis, our study results could still be biased by COPD severity. Further studies are required to evaluate the stages of COPD and NLR on short-term mortality for PE patients.

## Conclusions

NLR is an independent predicator for short-term mortality among PE patients. It enable sassessing the severity of PE, and could guide the clinical management of PE.
